# Preparation and Characterization of Super-Absorbing Gel Formulated from κ-Carrageenan–Potato Peel Starch Blended Polymers

**DOI:** 10.3390/polym13244308

**Published:** 2021-12-09

**Authors:** Mahmoud Moustafa, M. A. Abu-Saied, Tarek H. Taha, Mohamed Elnouby, Eman A. El Desouky, Saad Alamri, Ali Shati, Sulaiman Alrumman, Huda Alghamdii, Mohmed Al-Khatani, Rahmah Al-Qthanin, Ahmed Al-Emam

**Affiliations:** 1 Department of Biology, College of Science, King Khalid University, Abha 9004, Saudi Arabia; saralomari@kku.edu.sa (S.A.); aaalshati@kku.edu.sa (A.S.); salrumman@kku.edu.sa (S.A.); hudaghamdi@kku.edu.sa (H.A.); dr.malkahtani@gmail.com (M.A.-K.); rngerse@kku.edu.sa (R.A.-Q.); 2 Department of Botany and Microbiology, Faculty of Science, South Valley University, Qena 83523, Egypt; 3 Polymer Materials Research Department, Advanced Technology and New Materials Research Institute, City of Scientific Research and Technological Applications (SRTA-City), Alexandria 21934, Egypt; mouhamedabdelrehem@yahoo.com; 4 Environmental Biotechnology Department, Genetic Engineering and Biotechnology Research Institute (GEBRI), City of Scientific Research and Technological Applications (SRTA-City), Alexandria 21934, Egypt; t.h.taha@gmail.com; 5 Composite and Nanostructured Materials Research Department, Advanced Technology and New Materials Research Institute, City of Scientific Research and Technological Applications (SRTA-City), Alexandria 21934, Egypt; mnano2050@yahoo.com; 6 Chemistry Department, Faculty of Science, Alexandria University, Alexandria 21568, Egypt; emanawad473@gmail.com; 7 Prince Sultan Bin Abdulaziz Center for Environmental and Tourism Research and Studies, King Khalid University (KSA), Abha 62529, Saudi Arabia; 8 Department of Pathology, College of Medicine, King Khalid University, Abha 9004, Saudi Arabia; amalemam@kku.edu.sa; 9 Department of Forensic Medicine and Clinical Toxicology, Faculty of Medicine, Mansoura University, Mansoura 35516, Egypt

**Keywords:** κ-carrageenan/starch blend, hydrogel, instrumental analysis, biodegradability, children’s toys

## Abstract

κ-carrageenan is useful for its superior gelling, hydrogel, and thickening properties. The purpose of the study was to maximize the hydrogel properties and water-absorbing capacity of κ-carrageenan by blending it with starch from potato peels to be used as safe and biodegradable water-absorbent children’s toys. The prepared materials were analyzed using FTIR and Raman spectroscopy to analyze the functional groups. Results showed that there was a shift in the characteristic peaks of starch and κ-carrageenan, which indicated their proper reaction during blend formation. In addition, samples show a peak at 1220 cm^−1^ corresponding to the ester sulfate groups, and at 1670 cm^−1^ due to the carbonyl group contained in D-galactose. SEM micrographs showed the presence of rough surface topology after blending the two polymers, with the appearance of small pores. In addition, the presence of surface cracks indicates the biodegradability of the prepared membranes that would result after enzymatic treatment. These results are supported by surface roughness results that show the surface of the κ-carrageenan/starch membranes became rougher after enzymatic treatment. The hydrophilicity of the prepared membranes was evaluated from contact angle (CA) measurements and the swelling ratio. The swelling ratio of the prepared membranes increased gradually as the starch ratio increased, reaching 150%, while the water-uptake capacity increased from 48 ± 4% for plain κ-carrageenan to 150 ± 5% for 1:2 κ-carrageenan/starch blends. The amylase enzyme showed an effective ability to degrade both the plain κ-carrageenan and κ-carrageenan/starch membranes, and release glucose units for up to 236 and 563, respectively. According to these results, these blends could be effectively used in making safe and biodegradable molded toys with superior water-absorbing capabilities.

## 1. Introduction

Hydrogels are commonly used for bio-fabrication, as they are biocompatible materials [1,2]. Historically, natural hydrogels have been commonly used for tissue engineering, including alginate, collagen, gelatin, and κ-carrageenan [3]. κ-carrageenan is a generic name for a family of water-soluble, sulfated polysaccharides present in the cell walls of members of the Gigartinales and used extensively as gels and thickeners in the food industry. According to research on their biodegradability, they can be disintegrated and broken down into carbon dioxide and water by microorganisms under humid conditions [4]. They have a great potential to be used in the future as packaging materials, cutlery, diapers, etc. [5].

κ-carrageenan is a family of polysaccharides extracted from a specific red seaweed species in the Rhodophyceae family, formed by alternate units of D-galactose and 3,6-anhydro-galactose (3,6-AG) joined by 1,3 and β-1,4-glycosidic linkage. κ-carrageenan has the ability to form gels and membranes with better mechanical properties [6,7]. It has been known for its superior water-absorbing capacity, which allows it to be used for various medical [8], electrochemical [9], food, and environmental applications [10].

Starch is an inexpensive and readily available material that is produced from renewable resources and can be efficiently used [6,11,12]. It has been previously applied in many industries, such as pharmaceuticals, paper, textiles, and the food industry [13,14], and can be applied by many companies as a hydrogel material [15]. Among the various starch sources, wasted starch from potato peels is considered an economical source of starch [16], which could be used in many applications, such as enzyme production [17].

Starch comprises linear amylose and branched amylopectin structures. Amylose is composed of α-1,4 glucose units. This moiety supports the ability for film-forming. Starch-based films are often brittle and present weak mechanical properties. Therefore, starch can be mixed with other polymers to produce blends with excellent mechanical traits after solvent evaporation. Polymer blending is a well-used strategy whenever a change or improvement of properties is necessary. Starch-based films were created by blending starch with gelatin [18], lignocellulose nanofibers [19], chitosan [20], and biodegradable poly (vinyl alcohol) (PVA) [21].

Few studies have reported on starch/κ-carrageenan blends. κ-carrageenan has a linear sulfated structure obtained from red seaweed. It is composed of D-galactose and 3,6-anhydro-D-galactose units linked by α-1,3 and β-1,4-glycosidic linkages. κ-carrageenan has gelling and film-forming features. These traits allow the production of biodegradable films [22].

The aims of the present work were to develop blends composed of mixtures of κ-carrageenan and starch to make safe and biodegradable molded toys as a novel application. κ-carrageenan was combined at a high temperature with various percentages of starch in the presence of glycerol to form a viscous liquid solution that can be poured into a mold, cooled at room temperature till complete solidification, and finally reshaped using shaped molds (for example, animal-shaped molds). An evaluation was carried out by analyzing the films’ mechanical and swelling properties, these being the chemical interactions between the two polysaccharides, assessed by Fourier-transform infrared (FTIR) spectra, Raman spectra, and a scanning electron microscope (SEM). In addition, the hydrophilicity of the prepared membranes was evaluated from contact angle (CA) and water uptake (WU) measurements, and their potential biodegradability was also studied.

## 2. Materials and Methods

### 2.1. Materials

κ-carrageenan was obtained from Across Organics, and the α-amylase enzyme (700 U/mL) was purchased from Sigma-Aldrich(St. Louis, MO, USA). RPI Glycerol Liquid, USP grade, and molecular weight (92.1).

### 2.2. Extraction of Potato Peel Starch

The potato peels, which were collected from different restaurants in Borg El-arab city, were washed with tap water, then cut into 1 × 1 cm square pieces. The formed pieces were submerged in tap water and homogenized using a kitchen blender, then sieved using stainless-steel mesh (kitchen stainless steel sieve with filter sifter). The obtained large particles were discarded, while the starch-containing filtrate was left at room temperature till full evaporation of its water content. The dried starch molecules were collected, ground, and kept in a dry place till use.

### 2.3. Preparation of κ-Carrageenan and κ-Carrageenan/Starch Blend

**Scheme 1:**
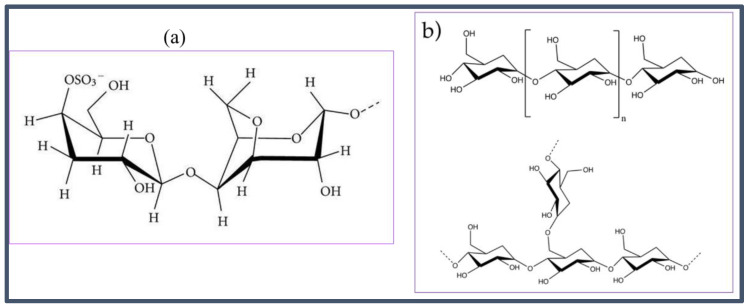
Chemical structure of κ-carrageenan (**a**) and starch (**b**).

**Figure 1:**
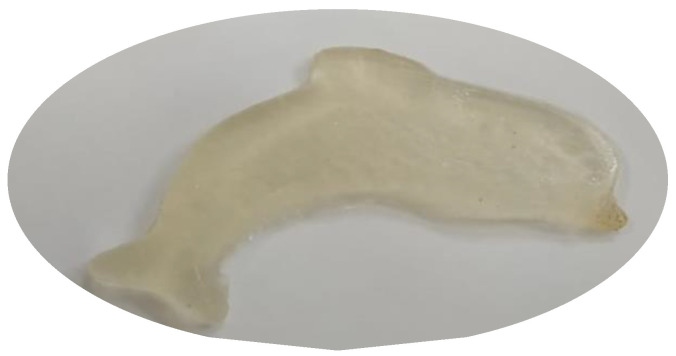
Dolphin gel formed by the blending of Carr/starch blend with superior water-absorbing capability.

κ-carrageenan solution (5%) was prepared by dissolving in boiling water. Starch solution was prepared by dispersing varying proportions (2.5–10%) of starch in distilled water. Blended solutions were prepared by mixing their pre-prepared solutions while stirring at 80 °C to obtain κ-carrageenan/starch blends at different ratios of 2:1, 1:1, and 1:2 with dropwise addition of glycerol (50 µm), which make membranes more elastic and enhances their mechanical characteristics. The prepared solutions were cast in a dolphin-shaped mold and kept at 27 °C for 48 h under 35% humidity (Figure 1). It is worth mentioning that dried membrane from each κ-carrageenan/starch blend was prepared in order to facilitate their characterization process. In addition, the chemical structure of κ-carrageenan and starch can be shown in Scheme 1.

### 2.4. Characterization

κ-carrageenan and κ-carrageenan/starch blend were characterized via several characterization methods, described as:

#### 2.4.1. Structural Characterization

The presence of the functional groups of both mixed and individual polymers was verified by Fourier-transform infrared (FTIR) technique (Shimadzu FTIR-8400 S, Japan) [23] and Raman scattering spectrometer (SENTERRA-Bruker, Ettlingen, Germany) [24]. The surface morphology of prepared κ-carrageenan, starch, and κ-carrageenan/starch membranes was observed using SEM (JEOL JSM-6360LA, Tokyo, Japan) [25]. Specimens of the resultant membranes were examined at an acceleration voltage of 10 KV. The surface roughness of all prepared membranes was determined using a surface roughness meter (SJ-201P, Kawasaki, Japan) [26,27].

#### 2.4.2. Water Uptake, Swelling Ratios, and Water Contact Angle

Water uptake of the prepared membranes was measured by the change in the weight between the wet ($W_{w})$ and dry ($W_{d})\;$ samples at 27 °C, as shown in the following equation [28].

$$WU=\frac{W_{w}-W_{d}}{W_{d}}\times 100$$

The swelling ratio of the membranes was estimated by the difference between the wet and dry dimensions of the length or thickness, which can be calculated by

(1) $$SR=\frac{L_{w}-L_{d}}{L_{d}}\times 100$$

where *L_w_* and *L_d_* are the length of the wet and dry membranes, respectively.

The hydrophilicity of the prepared membranes was evaluated from contact angle (CA) measurements [29]. For each sample, CA was measured at room temperature using an optical system comprised of a zoom video lens (Ramé-Hart Instrument Goniometer, model 500-F1, Netcong, NJ, USA) connected to a charge-coupled device (CCD) camera operated via software. Contact angle was determined using Image J software (1.47v) with the plug-in drop shape analysis. Small drops (~2 µL) were manually deposited using a precision microliter pipette [30,31].

The tensile strength of the polymer electrolyte membranes was measured using a Universal Testing Machine (Shimadzu UTM, Kyoto, Kyoto, Japan) at room temperature. The specimens were 50 × 10 mm. Measurements were carried out at a constant speed of cross heads movement 5 mm/minute. Three measurements were taken for each sample, and the mean values were considered [32].

#### 2.4.3. In Vitro Testing of Biodegradability

This test has been performed according to with some modifications. The biodegradability of the prepared membranes was investigated through the incubation of 0.07 g of each membrane with 100 µL amylase enzyme in 1400 µL acetate buffer (pH 5.5). After 72 h incubation at 30 °C and 150 rpm, SEM, Raman spectroscopy, and FT-IR analysis were investigated using the solid residuals of each membrane. On the other hand, the concentration of the liberated glucose units was determined spectrophotometrically using glucose kit (Bio-System, Barcelona, Spain).

## 3. Results and Discussion

### 3.1. FTIR Spectra

**Figure 2:**
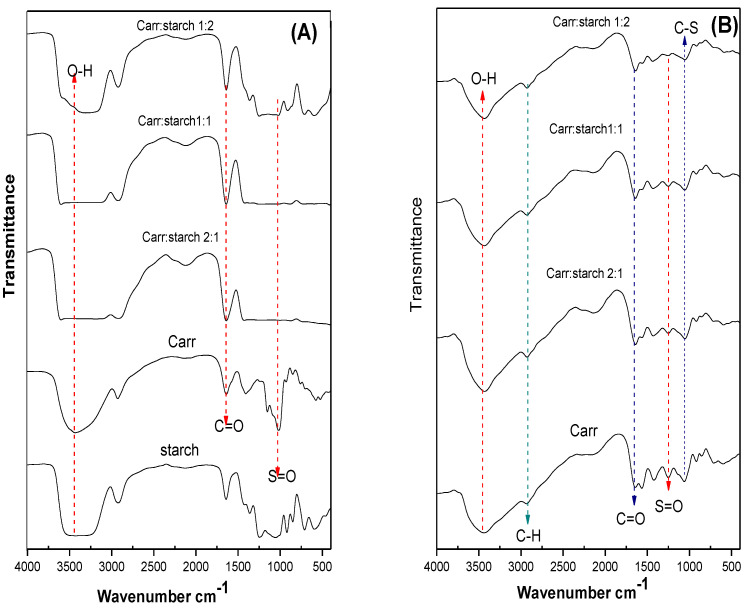
FTIR spectra of Carr, starch, and Carr/starch blend-based membranes before enzymatic treatment (**A**) and after enzymatic treatment (**B**).

Infrared spectroscopy is an effective method commonly used for polysaccharide characterization to study molecular interactions distinguished by the change of absorption bands. The fingerprint region of typical spectra of κ-carrageenan is presented in Figure 2A. The broad band ranging between 3500 and 3100 cm^−1^ was attributed to O-H stretching vibrations, formed due to the vibrational stretching associated with free inter- and intra- molecular bonds in -OH groups [19], and the broad band around 2800–3000 cm^−1^ was attributed to C-H stretching vibration. Samples also show a band in the region of 750–1300 cm^−1^ that corresponds to the carbohydrates region [17,33]. The peak at 1220 cm^−1^ corresponds to the ester sulfate groups, and the peak at 1670 cm^−1^ is due to the carbonyl group contained in D-galactose [33]. Additionally, the FTIR spectra of starch typically show an extremely broad band at 3387 cm^−1^, and the band at 2930 cm^−1^ is attributed to the O–H stretching and the C–H stretching vibrations, respectively [34], as shown in Figure 2A. Meanwhile, the spectrum of carr/starch blend-based membranes shows stretching at 1200–1300 cm^−1^. Shifting of bands in this region is due to interactions between the charged functional groups of the C-O stretching band and the C-O-H group band from 1162 cm^−1^ (κ-carrageenan) to 1151 cm^−1^ (κ-carrageenan/starch 2:1) that can be related to the interaction (possibly through hydrogen bridges) of the OH groups of starch with the κ-carrageenan structure [35]. On the other hand, as shown in Figure 2B, after treatment of κ-carrageenan/starch membranes with an amylase enzyme, distinct peaks were visible at 3190–3380 cm^−1^ (s), due to N-H stretching of secondary amides, and 2980 cm^−1^, due to C-H stretching of the amylase enzyme, in addition to the characteristic peaks of κ-carrageenan/starch membrane that appeared [36].

### 3.2. Raman Scattering Spectra

**Figure 3:**
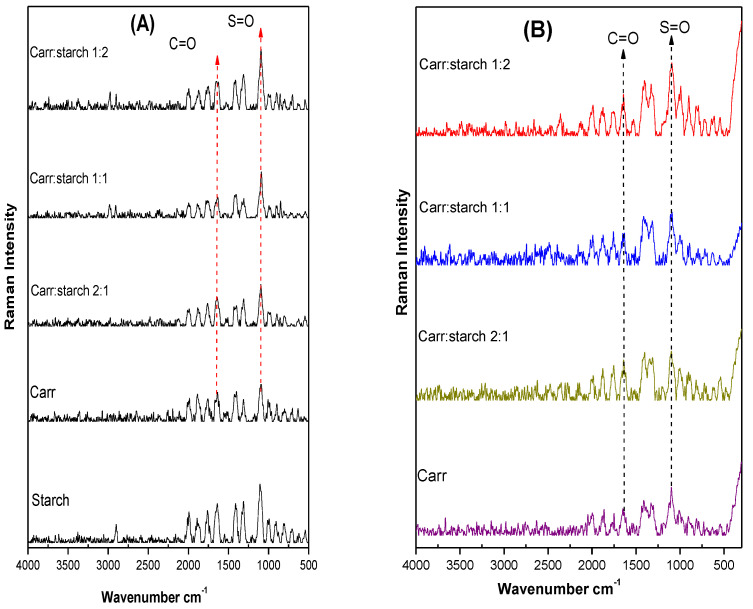
Raman scattering spectra of κ-carrageenan, starch, and Carr/starch blend-based membranes before (**A**) and after (**B**) treatment with amylase enzyme.

Raman spectroscopy results confirmed the interactions of starch with κ-carrageenan. Figure 3A shows the Raman spectra of starch, κ-carrageenan, and κ-carrageenan/starch membranes. The starch spectrum exhibited three characteristic scattering peaks at 1263, 1053, and 941 cm^−1^, which are due to the stretching of C-O, C-C, and C-OH bonds belonging to starch [37]. Absorption at 2812 cm^−1^ was due to the C-H stretching. Absorption at 1238 cm^−1^ was due to the ester sulfate group, and absorption at 1048 cm^−1^ was due to glycosidic linkage [38,39]. However, the κ-carrageenan/starch blend resulted in the appearance of a new peak at 1700 cm^−1^, which was due to the formation of the carbonyl group. This peak was initially thought to have resulted from the oxidation of carbohydrate radicals generated inside the κ-carrageenan polymer. However, as shown in Figure 3B, after enzymatic treatment, the characteristic peak of κ-carrageenan/starch membranes appeared as an additional peak at 3434 cm^−1^ due to NH_2_ stretching, which confirms the effect of the amylase enzyme on the degradation of the membranes [36,40].

### 3.3. In Vitro Biodegradability Test

**Figure 4:**
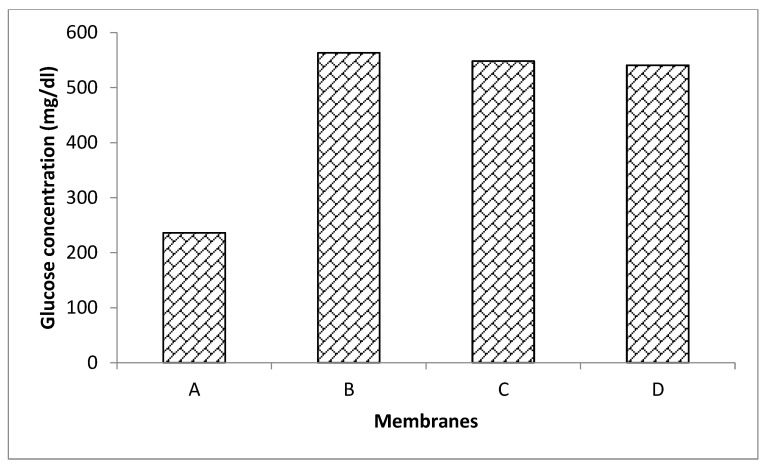
Concentration of glucose units released through the biodegradation of κ-carrageenan-starch membranes using amylase enzyme. (**A**): plain κ-carrageenan, (**B**): κ-carrageenan: starch (1:1), (**C**): κ-carrageenan: starch (2:1), and (**D**): κ-carrageenan: starch (1:2).

The investigation of the membranes’ biodegradability was dependent on the ability of the amylase enzyme to release glucose units from these membranes. As shown in Figure 4, higher concentrations of glucose were obtained from the three tested membranes (κ-carrageenan/starch 1:1, κ-carrageenan/starch 2:1, and κ-carrageenan/starch 1:2) in addition to lower glucose concentration from the plain κ-carrageenan membrane. The glucose concentration was almost 563, 548, and 540.5 mg/dL for the three mixed κ-carrageenan/starch membranes, respectively. However, the amylase enzyme showed an ability to degrade the κ-carrageenan polymer, and released 236 mg/dL glucose. In most cases, using pure enzymes for the biodegradation of waste is preferred over using whole living microbes due to their greater catalytic activity and the absence of seasonal fluctuations [41]. However, the use of soil microbes for the biodegradation of potato peel starch depending on hydrogel was investigated through soil burying of the prepared materials [42].

### 3.4. SEM Micrograph

**Figure 5:**
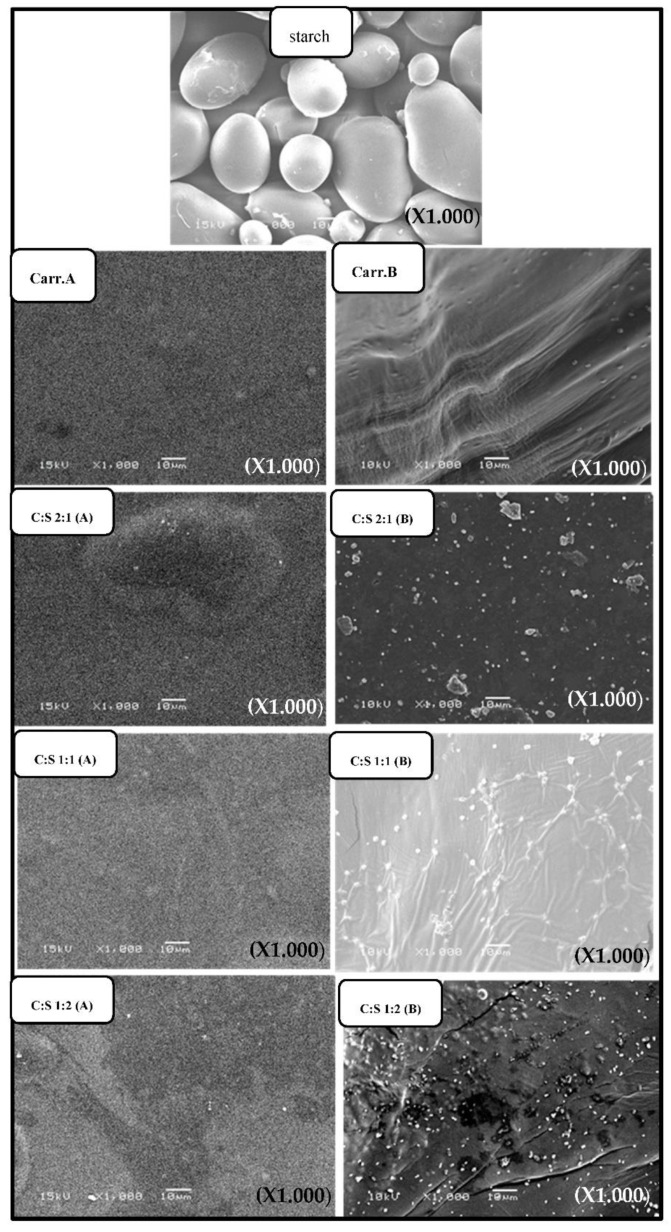
SEM micrograph of surface of starch, Carr, and Carr/starch blend-based membranes before (**A**) and after (**B**) enzymatic treatment at magnification power of 1000×.

SEM microphotographs of starch granules, as well as the surface of the κ-carrageenan/starch membranes are shown in Figure 5. The starch granules appeared oval, large, with compact and smooth surfaces [43]. On the other hand, the κ-carrageenan surface appeared as a homogenous membrane surface with no gross defects. Meanwhile, the presence of rough surface topology after blending starch and κ-carrageenan, and the appearance of small pores, indicate the presence of starch granules and the interaction of starch and κ-carrageenan. As represented in Figure 5A, membrane roughness proportionally increased as the concentration of starch was increased, and the membranes appeared without phase separation, which indicates a good interaction between the components, as confirmed by FTIR and Raman spectroscopy [44]. After enzymatic treatment, it was noticed that multiple cracks were shown on each membrane surface. These cracks grew as the starch concentration was increased, which indicates the potent ability of the enzyme to degrade the tested membranes. These data are strongly supported by the concentration of glucose that was released after the enzymatic treatment of the membranes (Figure 4). In addition, the existence of cracks indicates the membranes’ biodegradability.

### 3.5. Surface Roughness

Surface roughness values of the κ-carrageenan and κ-carrageenan/starch membranes with different ratios before and after enzymatic treatment are shown in Table 1. The surface roughness of the plain κ-carrageenan membrane was the lowest value (0.25 μm). This value was affected by blending κ-carrageenan with starch, especially κ-carrageenan/starch (1:2), which showed the highest value (1.1 μm). After enzymatic treatment, as represented in Table 1, the surface of the κ-carrageenan/starch membranes with different ratios increased in roughness, which indicates the effective degradation of the membranes by the amylase enzyme. These results are supported by the SEM images (Figure 5B).

### 3.6. Water Uptake and Contact Angle

Water uptake of the κ-carrageenan/starch membranes increased gradually as the starch ratio increased. As shown in Table 2A, the pristine κ-carrageenan showed the lowest swelling ratio, of around 48% [15], much lower than the starch-conjugated counterparts, which showed changes of between 70 and 150%. Furthermore, the high absorption of κ-carrageenan/starch could minimize the chances of phase separation, thus improving the permeability properties of the membrane. As shown in Table 2B, after enzymatic treatment, the swelling degree increased to reach 180% for the κ-carrageenan/starch (1:2) membrane. The increments of water uptake after the enzymatic treatment revealed the potency of amylase enzyme degradability, as the membrane degradation resulted in the increased porosity of the membranes’ surfaces, which allowed more water molecules to penetrate the matrix, resulting in an increased water uptake percentage. These data indicate the effect of the enzyme on the tested membranes and are good evidence for the potential of the prepared membranes as biodegradable plastic.

However, regarding the viscoelastic properties of the hydrogel: the gelation of κ-carrageenan has been widely studied using various methods, such as rheological experiments, to understand the properties of the mixed carrageenan gels.

The viscoelastic behavior of κ-carrageenan reveals a fluid-like character. At 9 °C, κ-carrageenan molecules are in the ordered state, but neither aggregation nor gelation takes place. Moreover, κ-carrageenan molecules being in the ordered state are obviously stiffer than in the disordered one.

In cases where κ-carrageenan is blended with starches, a synergistic effect on the rheological properties of the pastes and gels exists. Starch pastes and gels are considered a biphasic system, with the continuous phase consisting of the solvent and dissolved starch during gelatinization, and a disperse phase consisting of swollen granules. This viewpoint emphasizes the presence of swollen particles in starch suspensions. The swollen granules are not only deformable, but also compressible and elastic.

On the other hand, the hydrophilic/hydrophobic nature of the membranes’ surfaces is often indicated by the contact angle. Water contact angle values of the prepared membranes are listed in Table 3. Generally, the contact angle of κ-carrageenan membranes decreased with the addition of starch, from 84 θ to within a range of 70 to 60 θ (Table 3A). This decrement was more pronounced after enzymatic treatment [45]. The enzymatic treatment resulted in a decrease in the contact angle of κ-carrageenan to 70 θ. The contact angle was significantly decreased to between 60 and 35 θ with different starch ratios (Table 3B). According to these results, the κ-carrageenan/starch membranes can be effectively used as an improved hydrogel that can be projected to replace synthetic plastics.

### 3.7. Tensile Strength

The tensile strength and elongation at break for the prepared membranes are given in Table 4. When starch concentration in the blend formulation was increased, the tensile strength increased from 5.61 of plain Carr to 10.11 MPa at Carr/starch (1:1), which was recorded as the maximum measurement. However, the elongation at break decreased with the increasing concentration of starch. This could be due to the proper initiation of intermolecular interactions between the Carr polymer and the starch present in the system.

## 4. Conclusions

Blends of κ-carrageenan and potato peel starch were prepared in three different ratios. Both polymers were characterized before and after blending, using SEM, FTIR, Raman, etc., in addition to testing their biodegradability through enzymatic treatment. The results provide useful information on the structural properties of κ-carrageenan and starch and the structural changes in the network induced when mixing different ratios of the two polysaccharides, before and after enzymatic treatment. The FTIR spectra of κ-carrageenan/starch blend-based membranes showed stretching and shifting of different bands that can be related to their interaction. Additionally, SEM showed the presence of rough surface topology after the blending of starch and κ-carrageenan, indicating the presence of starch granules and the interaction of starch and κ-carrageenan. After enzymatic treatment, multiple cracks were noticed on each membrane surface, indicating potent enzymatic degradation of the prepared membranes. The swelling ratio and water contact angle results of the κ-carrageenan/starch membranes illustrate hydrophilic properties that increased gradually as the starch ratio increased. However, after enzymatic treatment, the swelling degree increased to reach 180% for the κ-carrageenan/starch (1:2) membrane due to the increased porosity of the membranes’ surfaces, which allowed more water molecules to penetrate the matrix. According to these results, it is proposed that κ-carrageenan/starch blends can be effectively used as a super-absorbent hydrogel that can be used as biodegradable children’s toys.

## Figures and Tables

**Table 1:** Surface roughness values of Carr/starch membranes before (A) and after (B) enzymatic treatment.

Sample	Roughness (μm) (A)	Roughness (μm) (B)
Carr	0.25 ± 0.02	0.50 ± 0.05
Carr: starch 2:1	0.49 ± 0.05	0.87 ± 0.03
Carr: starch 1:1	0.82 ± 0.04	1.11 ± 0.07
Carr: starch 1:2	1.1 ± 0.03	1.57 ± 0.08

**Table 2:** Water uptake and swelling ratio values of Carr/starch membranes before (A) and after (B) enzymatic treatment.

Sample	SR%(A)	WU% (A)	WU% (B)	SR% (B)
Carr	33.41 ± 2	48 ± 4	60 ± 5	40.21 ± 2
Carr: starch 2:1	40.14 ± 3	70 ± 2	78± 3	51.36 ± 1
Carr: starch 1:1	42.11 ± 2	90 ± 4	110 ± 2	60.11 ± 0.5
Carr: starch 1:2	54.35 ± 5	150 ± 5	180 ± 4	70.28 ± 1

**Table 3:** Water contact angle values of Carr/starch membranes before (A) and after (B) enzymatic treatment.

Sample	Mean Theta θ (A)	Mean Theta θ (B)
Carr	84 ± 3	70 ± 5
Carr: starch 2:1	70 ± 2	60 ± 5
Carr: starch 1:1	65 ± 4	45 ± 2
Carr: starch 1:2	60 ± 5	35 ± 2

**Table 4:** Mechanical properties of Carr and Carr/starch membranes.

Sample	Tensile Strength (MPa)	Elongation at Break (mm)
Carr	5.61 ± 2	5.34 ± 2
Carr: starch 2:1	7.24 ± 1	3.11 ± 1
Carr: starch 1:1	10.11 ± 3	2.01 ± 0.5
Carr: starch 1:2	4.35 ± 1	1.80 ± 1

## Data Availability

All data are available in the main text.
